# Supplementary figures and images for: From big data to diagnosis and prognosis: gene expression signatures in liver hepatocellular carcinoma
Source: PeerJ. 2017 Mar 14;5:e3089. doi: 10.7717/peerj.3089 (PMC5354077; doi:10.7717/peerj.3089)

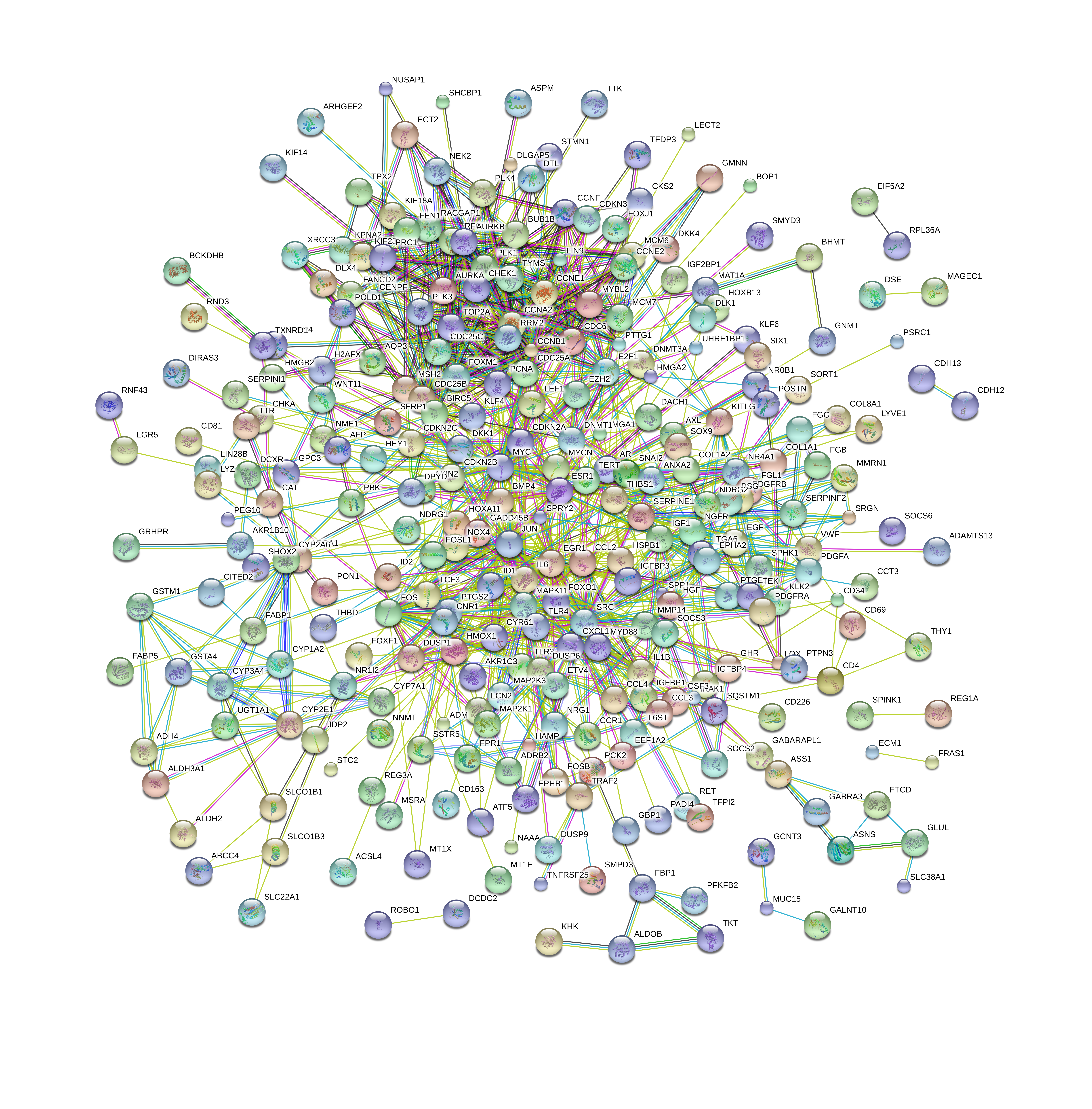

Supplement: Figure S1 — Interactions of all the DEGs were shown by STRING online database (http://string-db.org) with the cut-off criterion of combined score >0.7. Network nodes represented proteins and edges represented protein-protein associations. [file peerj-05-3089-s001.png]

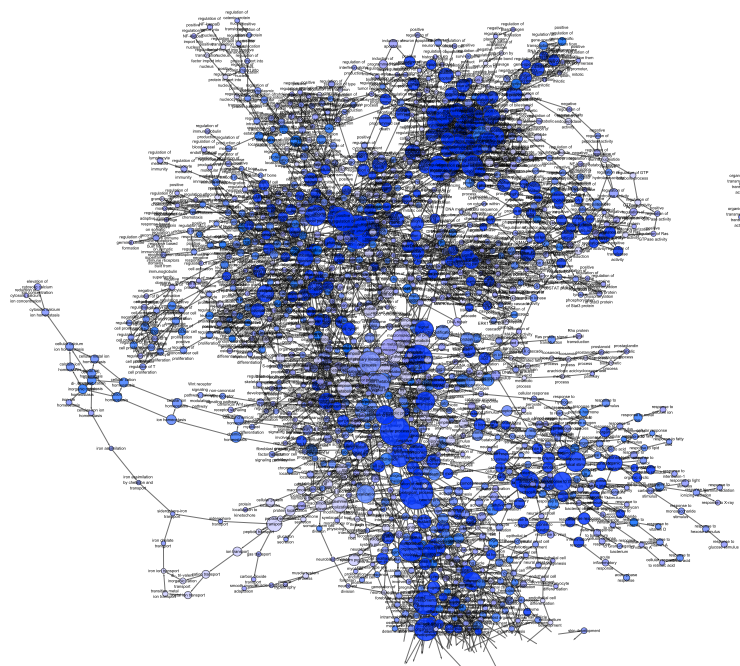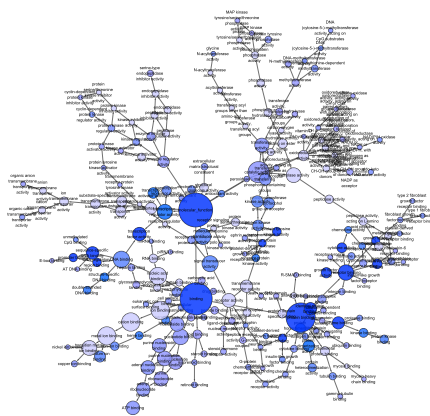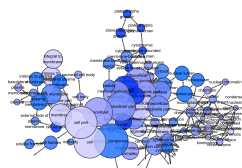

Supplement: Figure S2 — The circles represented different terms from the perspective of biological process (BP), cellular component (CC), and molecular function (MF). The relationships among terms were represented by arrows. A false discovery rate (FDR) of 0.05 was selected for the current directed acyclic graph (DAG). Altogether, 1583 nodes and 2676 edges were presented. The color depth indicated the significance of the corresponding term. [file peerj-05-3089-s002.pdf]

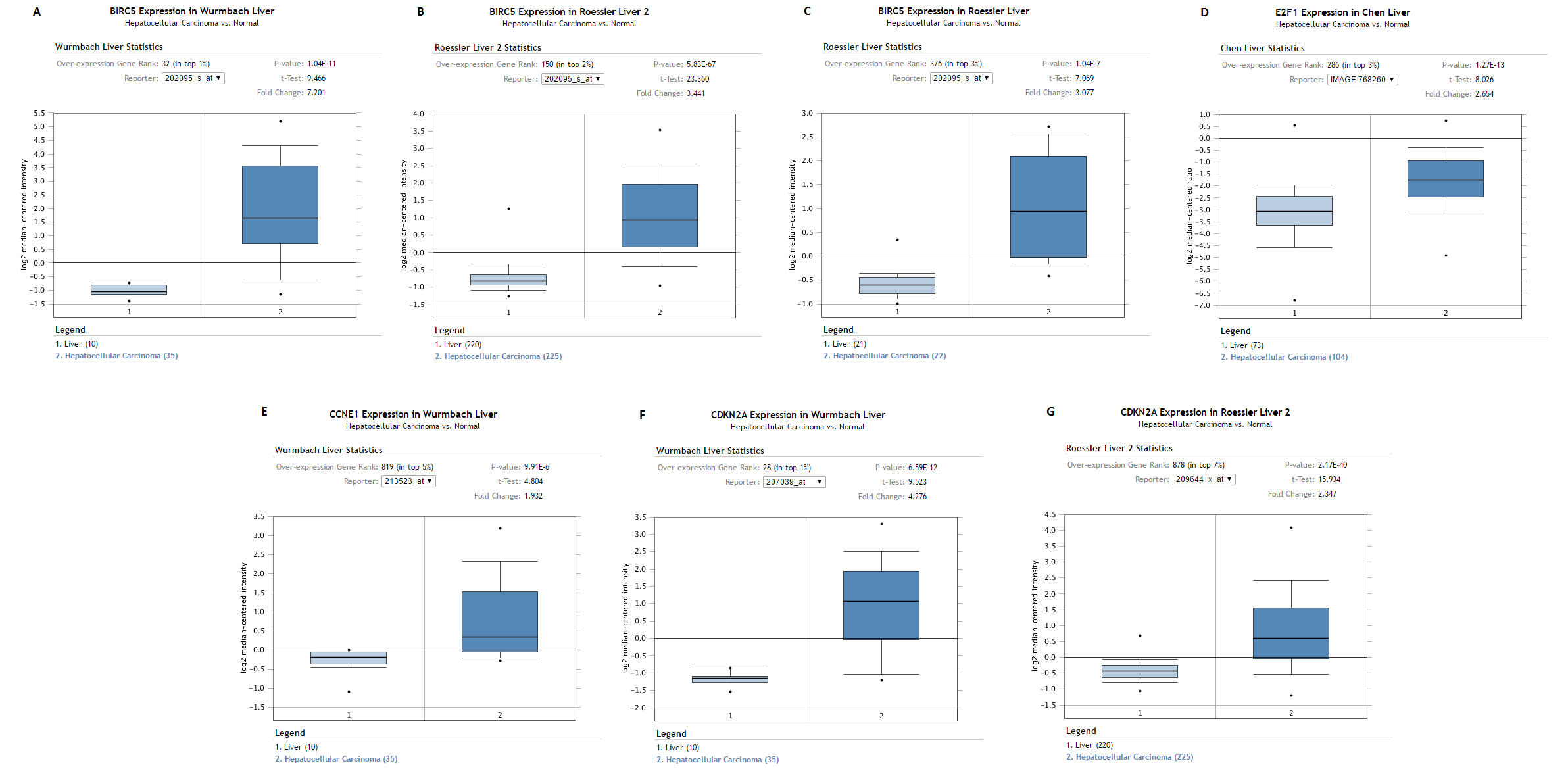

Supplement: Figure S3 — BIRC5, ECF1, CCNE1 and CDKN2A were searched in Oncomine Research Edition (https://www.oncomine.org) filtered with the Cancer Type of Liver Cancer. All these four genes were included in different datasets. Highly and reasonably consistent with the results from TCGA, all BIRC5 (Fig. A, B and C), ECF1 (Fig. D), CCNE1 (Fig. E) and CDKN2A (Fig. F and G) showed evidently over-expressed pattern in LIHC tissues. (A) BIRC5 expression between normal liver and LIHC tissues from Wurmbach Liver Statistics. Wurmbach Liver Statistics was based on the platform of Human Genome U133 Plus 2.0 containing 19,574 measured mRNAs. (B) BIRC5 expression between normal liver and LIHC tissues from Roessler Liver 2 Statistics. Roessler Liver 2 Statistics was based on the platform of Affymetrix Human Genome HT U133A Array containing 12,624 measured mRNAs. (C) BIRC5 expression between normal liver and LIHC tissues from Roessler Liver Statistics. Roessler Liver Statistics was based on the platform of Human Genome U133A 2.0 Array containing 12,603 measured mRNAs. (D) E2F1 expression between normal liver and LIHC tissues from Chen Liver Statistics. Chen Liver Statistics was based on the platform not pre-defined in Oncomine containing 10,802 measured mRNAs. (E) CCNE1 expression between normal liver and LIHC tissues from Wurmbach Liver Statistics. Wurmbach Liver Statistics was based on the platform of Human Genome U133 Plus 2.0 containing 19,574 measured mRNAs. (F) CDKN2A expression between normal liver and LIHC tissues from Wurmbach Liver Statistics. Wurmbach Liver Statistics was based on the platform of Human Genome U133 Plus 2.0 containing 19,574 measured mRNAs. (G) CDKN2A expression between normal liver and LIHC tissues from Roessler Liver 2 Statistics. Roessler Liver 2 Statistics was based on the platform of Affymetrix Human Genome HT U133A Array containing 12,624 measured mRNAs. [file peerj-05-3089-s003.png]
